# Monitoring the Attack Incidences and Damage Caused by the Almond Bark Beetle, *Scolytus amygdali*, in Almond Orchards

**DOI:** 10.3390/insects9010001

**Published:** 2018-01-01

**Authors:** Asma Zeiri, Muhammad Z. Ahmed, Andrew G. S. Cuthbertson, Mohamed Braham, Mohamed Braham

**Affiliations:** 1 Department of Biology, Faculty of Sciences of Bizerte, Bizerte 7021, Tunisia; 2 Florida Department of Agriculture and Consumer Services, Division of Plant Industry, 1911 SW 34th Street, Gainesville, FL 32614-7100, USA; 3 Independent Science Advisor, York YO10 5AQ, UK; andrew_cuthbertson@live.co.uk; 4 Laboratory of Entomology, Regional Center of Research on Horticulture and Organic Agriculture, The University of Sousse, Chott-Mariem, Sousse 4042, Tunisia; braham.mohamed@gmail.com; 5 Deparment of Olive Tree Physiology, Institute of the Olive Tree Station of Sousse, 40 Street Ibn Khouldoun, Sousse 4061, Tunisia; braham2015@yahoo.fr

**Keywords:** attack, almond orchard, distribution, feeding preference, Tunisia

## Abstract

The almond bark beetle, *Scolytus amygdali* Geurin-Meneville, is responsible for significant loss of fruit production in almond orchards throughout the world. Here, we studied the damage and the incidences of *S. amygdali* attack on two different scales: (1) at the level of a single tree; and (2) in an entire orchard. Our results revealed no differences in attack level among four orientations (east, west, south and north sides) for the whole tree. However, the bark that was facing west side in the direction of the prevailing wind was found to be the most suitable for females to initiate attack in Stratum S2. Attack distribution remains the same among different strata (strata is vertical divisions of the tree from the ground to the uppermost twigs with ~40 cm intervals). More than 50% of attack was observed in the trunk of the tree and upper strata. However, multiplication rate (number of emerged adults/maternal gallery) varies significantly between strata. In addition, we studied attack intensity (holes produced by beetle per tree) comparing it to tree morphology (flowers, leaves and circumferences) and gum deposit. Our results revealed a positive correlation between attack intensity and gum deposits, and a negative correlation between attack intensity and tree morphology. This revealed that gum on the tree was an indicator for attack intensity. A positive correlation between attack intensity and the circumference of the tree revealed that older trees were more susceptible to *S. amygdali* attack. These results, while preliminary, aim to help in the monitoring of *S. amygdali* populations before deciding to apply any control measures.

## 1. Introduction

Almond, *Prunus dulcis* (Rosaceae), is a tree native to the Middle East and South Asia. Almonds are an important component of human diets in many regions of the world, especially in the Mediterranean area. In Tunisia, the current area for the cultivation of almond trees is 228,000 ha in dry and 4500 ha in irrigated conditions [1]. The number of trees exceeds currently 27,500,000 with a good representation in the center of the country [1]. The fruit production contributes about 45% to the countries national agricultural production [2]. Tunisia is ranked eighth in the almond producing countries with an average of 66,700 tons per year [3]. Unfortunately, diseases and insect pests can damage almond production (Figure S1). Among them, Scolytid bark beetles are the most important [4]. *Scolytus amygdali* Geurin-Meneville, (Coleoptera: Curculionidae: Scolytinae), was identified as a major pest among bark beetles attacking almond in Tunisia especially in the center of the country [4,5].

*Scolytus amygdali* is generally a wood pest that attacks fruiting trees such as almond, peach, apricot, plum and apple [5]. These beetles target weak, old trees, where they make galleries and holes in the bark. This can destroy phloem tissues and disrupt the translocation of photosynthetic products which ultimately leads to death of the tree [6]. *Scolytus amygdali* is a predominant bark beetle species throughout the world that has been recorded as a pest of fruit trees since 1921 [6,7,8,9,10]. In Tunisia, it is a major pest, but it has been largely ignored by researchers due to the difficulty in studying both its biology and ecology. There have only been a few studies based on *S. amygdali* undertaken in Tunisia [4].

Female fecundity of *S. amygdali* varies with different ecological conditions [5,8,11]. Holes on infested trees represent a sign of emerging adults. Newly emerged adults from infested trees are attracted by chemical stimuli released by weak or stressed trees or other insect visual and olfactory stimuli, with the insect selecting between resistant and non-resistant hosts before colonization [8,11]. Once the pest begins feeding, it can attract other adults to the same location after initial copulation due to the release of aggregation pheromones [8,11]. Infested trees respond by releasing a gum defense (Figure S2). Holes left behind can weaken the tree even if it is not subsequently colonized. Beetle colonies establish once galleries get installed inside the bark further leading to the death of whole branches (Figure S2). Even though *S. amygdali* is considered as a secondary pest of fruit trees, they can kill branches, young trees or mature trees that have been weakened due to disease or water stress.

Pesticides such as deltamethrin and aggregation pheromones are often applied to control *S. amygdali*. Batta [12] introduced the entomopathogenic fungus *Beauveria bassiana* to control *S. amygdali*. In addition, cultural practices (e.g., pruning and burning of dead infested trees) are also frequently utilized [6,13,14]. Although these new control tactics have been introduced against *S. amygdali*, we are still lacking in understanding the attack strategies of this pest. Pest assessment is very critical for proper application of control measurements and the investigation of the attack strategies helps to assess the pest population in the field, which is in fact the prerequisite for designing any control applications for a given pest. Understanding how these beetles are distributed on the almond trees would help to investigate how, when and what control measurements (chemical, cultural or biological) should be applied. The aim of this study was to investigate the distribution of *S. amygdali* populations on an individual almond tree and in a whole almond orchard using orientation and strata as factors. Many aspects of the biology of *S. amygdali* are poorly understood and one of the hurdles in studying it is the fact that the beetle spends most of its life cycle under the tree bark. The best possible way to study its attack incidences is to cut down the whole infested tree and peel off its bark. However, each almond tree is economically very important, even if it is infested; therefore, depending on the intensity of damage, growers usually choose to treat before cutting down. In this study, we managed to locate only one heavily infested tree in the orchard and convinced the grower to let us cut it down, so that we could study the distribution and attack incidences of *S. amygdali* under the bark throughout the tree from its base to its top most twigs. In addition, we investigated beetle attack on the remaining trees in the whole orchard based on visual observations of *S. amygdali*’s attack to develop a scouting parameter against this pest.

## 2. Materials and Methods

### 2.1. Sampling Survey of S. amygdali Attack on an Almond Tree

**Figure 1:**
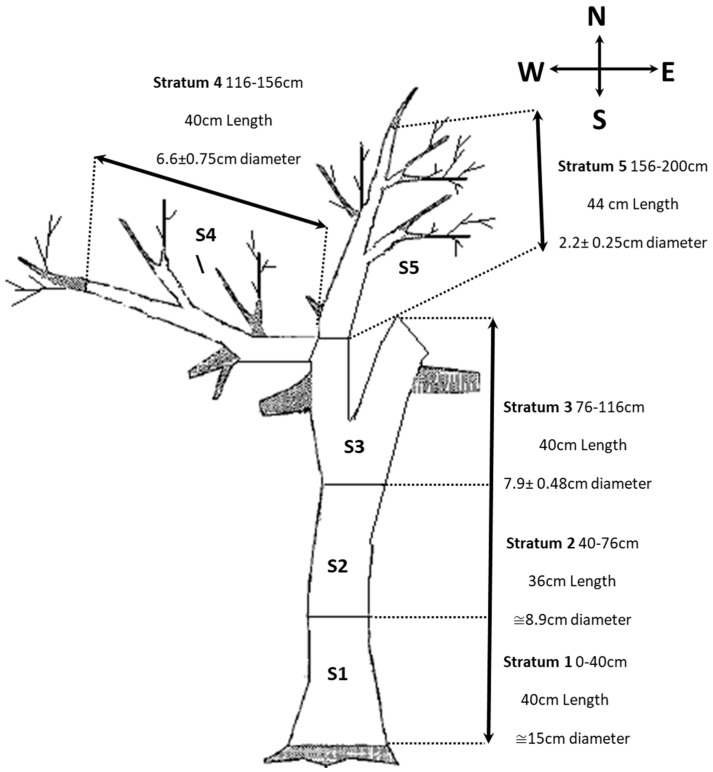
The vertical distribution of samples from an almond tree (Tree No. 47 as shown in Figure 2) used in this study.

**Figure 2:**
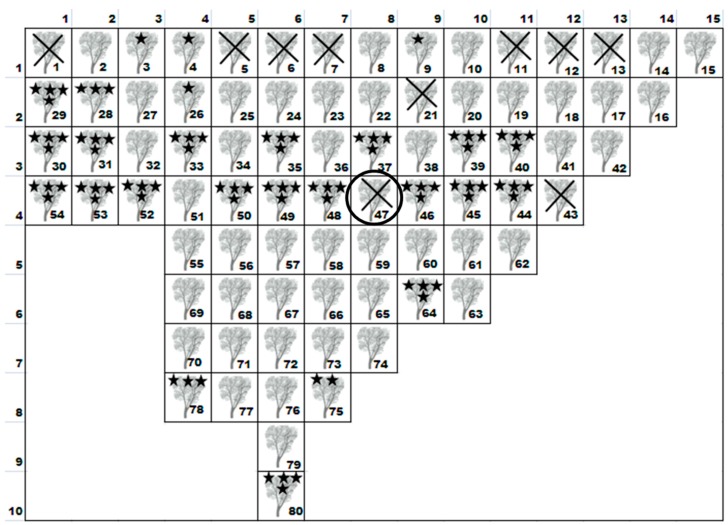
The layout of the almond tree orchard surveyed in this study; *X* and *Y* axis are number of trees. Crosses show those trees which were killed, dug out and burnt due to severe attack of *S. amygdali* except tree No. 47. Stars represent the attack intensity of *S. amygdali*: one star shows trees with an attack only on some twigs, two stars show trees with an attack on whole branches, three stars show trees with an attack up to one third of tree, and four stars show a tree which was completely attacked. The rest of the trees were found to be healthy. The circled tree was used in the study of vertical distribution, as shown in Figure 1.

This study focused on a single whole almond tree infested by *S. amygdali* in the middle of an orchard in Mahdia, a provincial center of Tunisia (35°19′60″ N, 10°25′0″ E) (Figure 1 and Figure 2). The study was conducted during February 2010. A dead, medium-size standing tree was chosen to evaluate the vertical distribution of the attack of the pest on the tree. The tree was cut down and divided into five sections called “strata” (i.e., vertical divisions of the tree at 40 cm intervals) from the ground to the uppermost twigs (S1 to S5) (Figure 1). The tree and strata were classified into four quadrants: east (E), west (W), north (N), and south (S) (Figure 1). Stratum S1 represents the trunk; Strata S2 and S3 represent further two ramified trunks connected with S1 and S4; and Stratum S5 represented branches: all were cut and organized using strata and orientated into groups. In total, 20 wood samples were brought back and analyzed in the laboratory. Each piece was measured for its length and circumference to determine its surface area. Enumeration of penetration holes of adult bark beetles were counted. We dissected each sample with a scalpel and used forceps to collect beetles. Maternal galleries tend to be single, longitudinal and made in an upward direction along the long axis of the attacked branch from the entrance hole [5]. Entrance tunnel shape can be different and each measure approximately 10.467 ± 3.24 mm in length [5]. All dead beetles found under the bark were collected to calculate the following parameters: (1) the number of maternal galleries—implying the attack number (AN)—which also represents number of females laying eggs (i.e., number of maternal galleries); (2) the number of maternal galleries in a cm^2^ area, which represents the attack density (AD); and (3) the number of emerged adults was determined by the number of maternal galleries representing the multiplication rate (MR), which also represented where the potential source of adult emergence was from on infested trees.

### 2.2. Survey of Almond Trees to Assess Scolytus amygdali Abundance/Density

We also measured the height, attack level of *S. amygdali*, the number of flowers, level of foliage cover and gum deposits on trees in the orchard to assess correlations between these variables and *S. amygdali*. The layout of the orchard is shown in Figure 2. At the start of the season in Tunisia (in March), a series of observations were made twice in 2010 on 80 random almond trees. Each tree was examined for the degree of attack using the following code notations: 0, no attack/no gum; 1, attack and/or gum on some twigs; 2, attack and/or gum on a whole branch; 3, attack and/or gum on less than two-thirds of the tree; 4, attack and/or gum on over two-thirds; and 5, attack and/or gum on the whole tree. These data were compared with the state of the foliage and flowering records at the same time using the following code notations: 0, no foliage or flowers; 1, a few leafy twigs (and/or flowers); 2, one leafy branch (and/or) flowers; 3, less than two-thirds of the tree leaves (and/or flowers); 4, leaves (and/or flowers) on more than two thirds, but not the entire tree; and 5, all the tree leaves (and/or flowers).

### 2.3. Data Analysis

Calculations of AN, AD and MR were conducted in MS Excel 2010. Pearson’s chi-squared test was conducted in IBM SPSS Statistics to analyze how the proportions vary between three parameters (AN, AD, and MR) by strata (S1–S5) and orientations (north, south, east, and west). The correlation between attack and the physiological status of the tree were analyzed by Spearman’s rho correlation coefficient. We also performed descriptive statistics of the orchard tree survey using Spearman’s rho correlation coefficient between attack intensity of *S. amygdali*, gum deposits on tree and morphological characteristics of the tree observed during the orchard tree surveys.

## 3. Results

### 3.1. Distribution of Scolytus amygdali Attack Abundance on a Tree

The total number of maternal galleries (AN) was 438 on the whole tree with a minimum of 11 (S4, north) and a maximum of 31 (S2, west). The sum of maternal galleries by orientation was 104 north, 112 east, 100 south and 122 west. The sum of maternal galleries by strata were 83 in S1, 110 in S2, 100 in S3, 82 in S4 and 63 in S5.

The results of Pearson’s chi-squared test (*X*^2^) between strata shows statistical difference for MR (*X*^2^ = 44.67, *df* = 12, *p* < 0.001), while the distribution of AN and AD remains the same along strata, (AN: *X*^2^ = 16.16, *df* = 12, *p* = 0.184; AD: *X*^2^ = 3.35, *df* = 12, *p* = 0.993) (Table 1). The attack number, AN, varies between a minimum of 14.38% at S5 and maximums of 22.83% and 25.11%, respectively, at S3 and S2 (Table 1). The attack density varies between a minimum of 7.02% at S1 and a maximum of 38.59% at S5 (Table 1). The multiplication rate varies between 12.19% at S1 and 26.87% at S3 (Table 1).

The results of Pearson’s chi-squared test (*X*^2^) between orientations and strata show only statistical difference in Stratum S2. The most registered attack (including attack number, attack density and multiplication rate) was in west (*X*^2^ = 0.99, *df* = 6, *p* = 0.040) of Stratum S2 (Table 1). Three out of the four strata have highest attack observed in the west without statistical difference (S1: *X*^2^ = 0.99, *df* = 6, *p* = 0.369; S3: *X*^2^ = 0.99, *df* = 6, *p* = 0.062; S4: *X*^2^ = 0.99, *df* = 6, *p* = 0.147; S5: *X*^2^ = 0.99, *df* = 6, *p* = 0.497) (Table 1). By orientation, AN varied between a minimum of 13.41% north (S3) and a maximum of 32.92% south (S4) (Table 1). In the case of AD, the highest level of maternal galleries per cm^2^ was 40% in south (S4) and a minimal level of 10% in the north (S4) (Table 1). A maximum MR was also recorded in the west (S1) with 41.70% and a minimum of 4.069% in the south (S2) (Table 1).

### 3.2. Survey of Almond Trees to Assess the Abundance of Scolytus amygdali Populations

We observed 80 trees in total within the orchard. Among them, 51.72% were healthy, 4.59% had attack only on some twigs, 1.14% had attack only on branches, 2.29% of trees had attack on less than two-thirds of the tree, and 40.22% had attack all over. In addition, there were ten trees that were completely removed and burnt previously by grower from the orchard because they had been killed due to *S. amygdali* attack (Figure 2).

Descriptive statistics of the orchard tree surveys showed that the circumference of the almond trees varied between 1.2 and 95 cm with a mean of 56.45 ± 17.91. Length of trees varied between 1.2 and 5 m with a mean of 2.34 ± 0.78. The correlation between attack intensity of *S. amygdali* and gum deposits was positive (*r* = 0.312, *p* = 0.009). A negative correlation was observed between attack intensity and flowering on trees (*r* = −0.488, *p* ≤ 0.001). Similar results were also observed for the presence of foliage (*r* = −0.309, *p* = 0.009). The correlation between attack intensity and the circumference of the trees was positive (*r* = 0.315, *p* = 0.008) (Table 2 and Table 3).

## 4. Discussion

In our study, the orientations (north, south, east and west sides) in respect to the whole tree showed no statistically difference for attack incidences in general. Benazoun [11,15] recorded similar results in Morocco. *Scolytus amygdali* can fly in any direction without preference; however, in our study, the orientation for various strata of a tree may vary and our results revealed that the west facing side was more suitable for female beetles to lay eggs in basal strata (S2). This might be explained by the location of the region where the survey was conducted (Costal zone of Mahdia, Tunisia): in this region, the wind direction in the spring generally blows towards the west, which would then probably stimulate flights and movements of beetles and might help them to attack the western strata of the tree [16]. Those results can also be explained by the dry cold weather in the region of Mahdia and the stress that it may cause on old trees [17]. Zeiri et al. [17] has also reported in a previous study that in this region the climate parameters had an important effect on flying pattern of *S. amygdali*. The distribution of attack incidences along strata vary significantly only in terms of multiplication rates. Benazoun [11,15] reported that *S. amygdali* on almond trees in Morocco has no preference for orientation. The distribution of incidences of attack for *S. amygdali* was statistically the same along strata, however more than 50% of AN was concentrated in the trunk of the tree (S1 + S2 + S3). Those results were also reported in other studies on almond in Morocco and *Cedrus atlantica* in Algeria [15,18]. Talbi [18] found the attack of *S. amygdali* was usually higher on the trunk and lesser on towards the smaller branches. Svihra [19] also found similar results for another bark beetle, *S. multistriatus*, on Chinese elms in China. In our study, about 40% of AD was observed in upper strata (S5) this is can be explained by the higher number of beetle families and the little surface available. Benazoun [11,15] found that mating systems were concentrated in basal and upper strata (S5). Upper strata present favorable conditions for feeding and mating, thin bark/foliage and males flying easily which assure a better larvae development [15]. In our study, the number of adult emerged/maternal galleries (MR) was higher in the middle of the tree in Strata S4 and S5 with a diameter that ranged between 6.6 ± 0.75 and 7.9 ± 0.48 cm. Talbi [18] revealed that adults of *S. amygdali* mostly emerge at the level of branches with a diameter range from 3.5 to 7 cm. Females of *S. amygdali* attack at basal and upper strata (S2, S3, and S5), whereas there was a higher proportion of adult emergence in the upper strata (S3 and S4). This may be explained by the behavior of larvae as they move away from maternal galleries in the search for food and so pupate far from their maternal galleries. The lowest attack observed in S1 was probably due to the thickness of the bark on the base. According to Chararas [20], most little beetles prefer branches with thin bark.

Although *S. amygdali* has shown some preference in concentration of attack within strata and orientation, those differences were not significantly different. The pattern of attack by *S. amygdali* might be affected by various conditions. In initial phase, females search for suitable trees depending on many factors like tree age, weakness, chemistry. Chemical compounds play an important role in *Scolytus* pheromone production and attraction [14]. Females are first to attack trees, after that they start sending out pheromone signals for more beetles to join and initiate the aggregation on the host tree [14]. This behavior can explain the pattern of attack in our study.

There is no comprehensive study for correlation between attack intensity of *S. amygdali* and almond tree morphological characters. We found a negative correlation between attack intensity and tree morphology (foliage and flowerings status). Our results are in agreement with the previous studies of Benazoun [11,15]; however, in our study, the negative correlation was stronger than that revealed by Benazoun [11,15]. A stronger negative correlation between attack intensity and flowering on trees here determines that flowering reduces greatly on a tree when the tree gets attacked by *S. amygdali*. This situation leads the tree towards weak health and eventually to its death. Benazoun [11,15] did not find any correlation between gum deposit on a tree and beetle attack intensity. However, we found a strong correlation between gum deposits and attack intensity of the beetle. A positive correlation between attack intensity and gum deposit determines that gum deposits on a tree are a clear indication of the attack intensity level on a given tree. Benazoun [11,15] also found that trees with a large circumference were attacked more, and we confirmed this in our study. Mahhou and Dennis [21] and Mendel [6] found similar results, in that older trees are more susceptible to beetle attack.

The presence of gum deposits particularly on the second stratum of the tree is a clear indication of serious *S. amygdali* attack. If the gum was observed only on some twigs or branches in Strata S4 or S5, it may indicate that the beetles might have just emerged and would be at a feeding stage, and may soon start looking for other suitable hosts. At this stage, the growers should start pruning infested/dead branches, and start taking care of weak and stressed trees. If the gum covers more than two-thirds of the tree and/or the attack density in Strata S2 and S3 are higher, then it should be assumed that we are facing an attack caused by females that may already have found maternal galleries successfully, and have started laying eggs. At this stage, it is suggested to apply chemical treatments on the whole tree. The west facing side in Stratum S2 was seen to be the most favorable for emergence of beetles and it therefore should be treated well during chemical application. If infestation is not observed at an early stage and gum is noticed all over the tree, then the tree should be dug out and discarded from the orchard before the emergence of adult beetles. It should also be noted that, when there has been a constant increase in the number of holes per surface area in S3 and S4, the removal of that tree may also be very effective in discarding an upcoming pest population attack. The almond bark beetle in the orchard of Souassi usually has three generations per year: the overwintering generation (November to January), a spring generation (March to April) and a summer generation (May to June) [5]. In these months, growers should perform pest scouting regularly and apply control measurements based on the above-mentioned observations. The population dynamics of *S. amygdali* (life cycle, number of generation, and reproduction) happen in a spatial and temporal framework and its attack could extend from a few centimeters squared to several kilometers squared, and could take between a few minutes to hours for adults to settle down on a tree [15]. Our study is useful in determining the criteria for an assessment and in planning the action threshold against *S. amygdali* attack in almond orchards. All of this may help in managing *S. amydali* populations within the orchards.

## Figures and Tables

**Table 1:** Pearson’s chi-squared test analysis to test the effect strata and orientations on attack incidences (attack number, attack density and multiplication rate) of *Scolytus amygdali*.

Strata	Orientations	Percent Attack Number (Total)	Percent Attack Density (Total)	Percent Multiplication Rate (Total)	Pearson Chi-Square
Stratum S1	North	22.89 (83)	25.00 (0.04)	08.30 (44)	*X*^2^ = 0.99, *df* = 6, *p* = 0.369
	South	25.30 (83)	25.00 (0.04)	36.92 (44)	
	East	20.48 (83)	25.00 (0.04)	13.06 (44)	
	West	31.32 (83)	25.00 (0.04)	41.70 (44)	
	**Total**	**18.95 (438)**	**7.02 (0.57)**	**12.19 (358)**	
Stratum S2	North	27.27 (110)	27.27 (0.11)	22.75 (72)	*X*^2^ = 0.99, *df* = 6, *p* = 0.040
	South	20.90 (110)	18.18 (0.11)	4.069 (72)	
	East	23.63 (110)	18.18 (0.11)	37.58 (72)	
	West	28.18 (110)	36.36 (0.11)	35.58 (72)	
	**Total**	**25.11 (438)**	**19.30 (0.57)**	**19.94 (358)**	
Stratum S3	North	26.00 (100)	20.00 (0.1)	27.88 (97)	*X*^2^ = 0.99, *df* = 6, *p* = 0.062
	South	14.00 (100)	20.00 (0.1)	32.61 (97)	
	East	30.00 (100)	30.00 (0.1)	19.92 (97)	
	West	30.00 (100)	30.00 (0.1)	19.57 (97)	
	**Total**	**22.83 (438)**	**17.54 (0.57)**	**26.87 (358)**	
Stratum S4	North	13.41 (82)	10.00 (0.1)	27.48 (85)	*X*^2^ = 0.99, *df* = 6, *p* = 0.147
	South	32.92 (82)	40.00 (0.1)	18.47 (85)	
	East	29.26 (82)	30.00 (0.1)	25.097 (85)	
	West	24.39 (82)	20.00 (0.1)	28.94 (85)	
	**Total**	**18.72 (438)**	**17.54 (0.57)**	**23.70 (358)**	
Strataum S5	North	28.57 (63)	27.27 (0.22)	24.38 (62)	*X*^2^ = 0.99, *df* = 6, *p* = 0.497
	South	23.80 (63)	22.72 (0.22)	13.44 (62)	
	East	23.80 (63)	22.72 (0.22)	20.97 (62)	
	West	23.80 (63)	27.27 (0.22)	41.19 (62)	
	**Total**	**14.38 (438)**	**38.59 (0.57)**	**17.28 (358)**	
Pearson chi-square		*X*^2^ = 16.16, *df* = 12, *p* = 0.184	*X*^2^ = 3.35, *df* = 12, *p* = 0.993	*X*^2^ = 44.67, *df* = 12, *p* < 0.001 *	

*X*^2^: Pearson’s cumulative test statistic; *df*: the number of freedom; *p*: probability; Total: the sum of the values of each parameter (attack number, attack density and multiplication rate) in all five strata; * 0.05.

**Table 2:** Descriptive statistics of the trees height and their circumference taken during the orchard tree survey.

	N	Min.	Max.	Sum	Mean	SD
Height	70	1.2	5.0	164	2.343	0.7876
Circumference	70	24	95	3952	56.45	17.913

SD: Standard Deviation.

**Table 3:** Correlations between attack intensity of *S. amygdali*, gum deposits on tree and morphological characteristics of the tree observed during the orchard tree survey.

	Gum	Foliar	Flower	Circumference
Attack intensity	0.312 **	−0.309 **	−0.488 **	0.315 **
	0.009	0.009	<0.000	0.008

** 0.01.

## Supplementary Materials

The following are available online at www.mdpi.com/2075-4450/9/1/1/s1, Figure S1: Trees infested by *S. amygdali*: (a) partially infested; and (b) completely infested tree, Figure S2: (a,b) Damage of *S. amygdali* gum deposits on a tree; and (c,d) tunnel holes on a tree.
