# Supplementary material for: Monitoring the Attack Incidences and Damage Caused by the Almond Bark Beetle, Scolytus amygdali, in Almond Orchards
Source: Insects. 2018 Jan 1;9(1):1. doi: 10.3390/insects9010001 (PMC5872266; doi:10.3390/insects9010001)
Supplement: Supplementary file 1 [file insects-09-00001-s001.pdf]

insects

Article

Monitoring the Attack Incidences and Damage Caused by the Almond Bark Beetle, *Scolytus amygdali*, in Almond Orchards

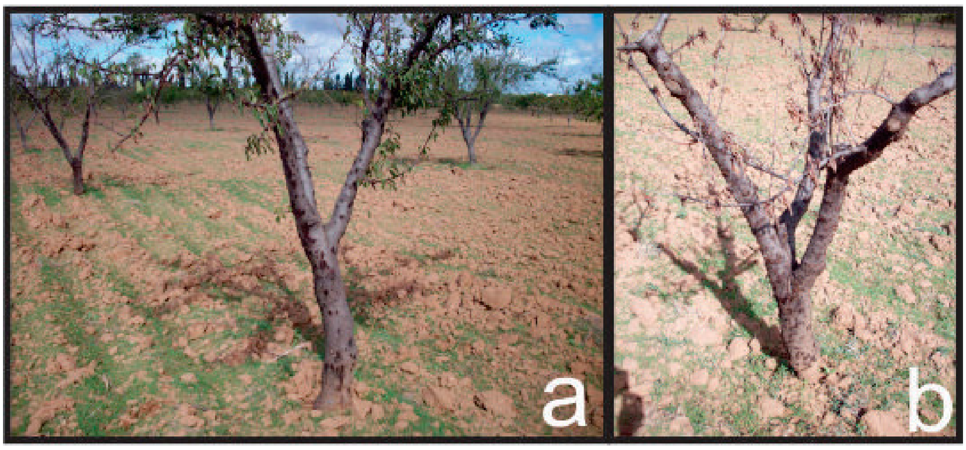

Figure S1. Trees infested by *S. amygdali*, (**a**) Partially infested; (**b**) completely infested tree.

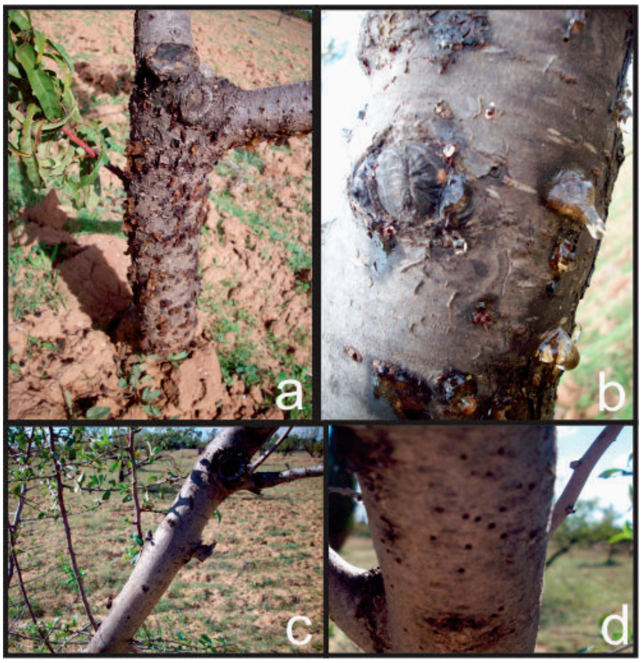

Figure S2. (**a**,b)—damage of *S. amygdali* gum deposits on a tree; (**c**,**d**)—tunnel holes on a tree.
